# Changes in the Metabolome and Nutritional Quality of Pulp from Three Types of Korla Fragrant Pears with Different Appearances as Revealed by Widely Targeted Metabolomics

**DOI:** 10.3390/plants12233981

**Published:** 2023-11-27

**Authors:** Wei Jiang, Pan Yan, Qiangqing Zheng, Zhendong Wang, Qiling Chen, Yi Wang

**Affiliations:** 1 Xinjiang Production and Construction Corps, Key Laboratory of Korla Fragrant Pear Germplasm Innovation and Quality Improvement and Efficiency Increment, Xinjiang Academy of Agricultural and Reclamation Sciences, Shihezi 832061, China; 15329524602@163.com (W.J.); yanpan11235@163.com (P.Y.); zhengqq369@163.com (Q.Z.); wzdanjing@163.com (Z.W.); 2 College of Horticulture and Landscape Architecture, Zhongkai University of Agriculture and Engineering, Guangzhou 510225, China

**Keywords:** Korla fragrant pear, UPLC–MS/MS, metabolomics, convex calyx

## Abstract

Korla fragrant pear (*Pyrus sinkiangensis* Yü) fruits have a unique flavor and are rich in phenolic acids, flavonoids, amino acids, and other nutrients. At present, the molecular basis of the quality differences among Korla fragrant pear fruits with a convex calyx and rough skin (RS), calyx shedding (SD), and a convex calyx (CV) remains unknown. To analyze the main metabolic components of Korla fragrant pear fruits and compare the antioxidant activities of these three fruits with different qualities, we used nutrient composition analysis and ultra-high-performance liquid chromatography-tandem mass spectrometry (UPLC–MS/MS)-based widely targeted metabolomics approaches to analyze the changes in the quality characteristics of the pulp of these three Korla fragrant pear fruits with different appearances. The nutrient composition analysis showed that the fructose and glucose contents were not significantly different, and sucrose and vitamin C contents were significantly higher in SD fruits compared with CV and RS fruits. However, the levels of flavor substances such as titratable acids, total phenols, and total flavonoids were high in the pulp of RS fruits. The metabolomics results identified 1976 metabolites that were clustered into 12 categories, and phenolic acid and flavonoid metabolites were the most abundant. The differentially accumulated metabolites (DAMs) in the fruits with different appearances were screened by multivariate statistical methods, and a total of 595 DAMs were detected. The analysis identified 300 DAMs between the CV and SD fruits, 246 DAMs between the RS and CV fruits, and 405 DAMs between the RS and SD groups. SD fruits contained the most metabolites with a high relative content, especially phenolic acids, lipids, amino acids and derivatives, alkaloids, and organic acids. Compared with CV fruits, flavonoid metabolism was more active in RS fruits, which also had a higher content of flavonoids, whereas the fewest metabolites were found in CV fruits, which also displayed less flavonoid accumulation. KEGG pathway enrichment analysis revealed that the DAMs were mainly enriched in the metabolic pathways of flavone and flavonol biosynthesis, confirming that CV fruits have decreased flavone and flavonol biosynthesis and accumulate fewer flavonoids than RS fruits, which may explain the less bitter and astringent flavor of CV fruits. However, the flavonoid content in RS fruits was very high, which may be one of the reasons why RS fruits have a harder pulp and are less juicy, more slaggy, and less flavorful. Moreover, the analysis of the antioxidant activity showed that during fruit development and maturation, RS fruits had stronger antioxidant activity than SD and CV fruits. These results provide a theoretical basis for improving the fruit quality of Korla fragrant pears and the processing of pear pulp.

## 1. Introduction

Korla fragrant pear (*Pyrus sinkiangensis* Yü) is an endemic economic forest species in China that is mainly distributed in the southern Xinjiang region and has become a high-end, characteristic fruit tree species grown on a large scale in this region [1,2]. Korla fragrant pears fruits are rich in nutrients and contain a variety of mineral elements because of their thin exocarp, crispy flesh, high juice and sugar content, pleasant, rich fragrance, and popularity among consumers [3]. Compared with other Chinese main pear varieties (e.g., Yali pear, Dangshan pear, Jingbai pear, and Nanguo pear), Korla fragrant pear has higher glucose and sucrose contents, and its fructose content is close to that of Nanguo pear [4]. Research has shown that the fragrant pear, compared with other pear varieties, has a relatively low organic acid content that is mainly malic acid. The contents of sugars and organic acids in Korla fragrant pears are significantly higher than those in Yali pear, Dangshan pear, Jingbai pear, and other main planted pear varieties. Therefore, the high sweetness and low organic acid content of Korla fragrant pears are the basis of their high quality and are key characteristics that distinguish them from other pear varieties [4,5].

In *P. sinkiangensis* Yü, the fruits that are fusiform with a protruding calyx end that is persistent are called calyx convex pears, whereas the fruits that are ovoid with a top that is concave like a funnel and no calyx are called calyx shedding pears. Fruits with a shed calyx have higher contents of soluble sugars and vitamin C and lower contents of titratable acids compared with fruits with a convex calyx [6]. Persistent calyx fruits have the characteristics of an inconsistent fruit shape, more stone cells, large kernels, a small edible portion, a light flavor, and acidity near the kernel, which seriously reduces the economic value of Korla fragrant pears. Among the convex calyx fruits, there are also those that will become rough-skinned with a calyx present. Extensive and in-depth research on fruit calyx shedding and calyx convexity has been carried out with Korla fragrant pears [6,7,8], the affecting factors [9], and the regulatory measures [10,11]. Recently, studies of Korla fragrant pears have focused on calyx abscission, which is significantly affected by auxins. High content of abscisic acid promoted calyx abscission, whereas high content of indole-3-acetic acid and gibberellic acid are detrimental to abscission [12]. The study indicated that spraying paclobutrazol (PP_333_) was effective in increasing calyx abscission rates [13]. Furthermore, the pollination variety also significantly affects the calyx abscission rate of Korla fragrant pear fruits, and research results have confirmed that the calyx abscission rate was higher after pollination with Xueqing, Yali, Zhonglihao, and Cuiguan pears [14]. With the continuous expansion of the Korla fragrant pear cultivation area, the yield has increased substantially; however, fruit quality has declined, and the proportions of convex calyx fruits and rough-skinned fruits have increased. Korla fragrant pear fruits with poor appearance usually have the shortcomings of calyx protrusion, an unattractive appearance, and rough skin. The fruits with convex and rough-skinned calyxes not only have a poor appearance but also hard pulp and a reduced sugar content, often exhibiting characteristics of less juice and more slag, which seriously affect the economic value of Korla fragrant pear and reduce its market competitiveness. However, the molecular basis of the quality differences among pear fruits with convex, shedding, and rough-skinned calyxes remains unknown. Thus, classifying the fruit quality, processing, and utilizing poor-quality fruits are important for improving the industrial value of Korla fragrant pears.

Metabolomics is the study of endogenous metabolites in organisms, including organic acids, amino acids, lipids, sugar alcohol compounds, and other substances, analyzing small-molecule metabolites with a molecular mass of less than 1000 [15]. At present, a wide range of targeted metabolomics technologies based on ultrahigh-performance liquid chromatography-tandem mass spectrometry (UPLC–MS/MS) with excellent chromatographic separation, high sensitivity, and high resolution have been widely used in many fields, such as food science, fruit development, and the evaluation of endogenous plant components [16,17,18,19]. Metabolomics studies on *Arabidopsis thaliana* and *Coreopsis tinctoria* from different regions revealed that the growth environment significantly affected the contents and types of amino acids and sugars in *A. thaliana* and that the flavonoid composition in *C. tinctoria* grown at high altitude was significantly different from that of *C. tinctoria* grown at low altitude [19,20]. To reveal the diversity and germplasm-specific flavonoids in radish, the metabolic profiles in the skin and flesh of six colored radish germplasms were analyzed by liquid chromatography–electrospray ionization tandem mass spectrometry. The results revealed that there were significant differences in the flavonoid metabolites among radishes with different qualities, and a total of 133 flavonoids, including 16 dihydroflavones, 44 flavones, 14 flavonoids, nine anthocyanins, and 28 flavonols, were identified [21]. Wang et al. [22] analyzed the metabolites of *Lycium barbarum* in Ningxia using UPLC–MS/MS technology and found that the types and contents of metabolites of *L. barbarum* from different origins varied greatly and that external factors, such as temperature and altitude, also altered the types and contents of metabolites in *L. barbarum* to different degrees.

In this study, we used nutrient composition analysis and UPLC-MS/MS-based widely targeted metabolomics approaches to analyze the changes in the quality characteristics of *P. sinkiangensis* Yü pear fruits with a convex calyx and rough skin (RS), calyx shedding (SD), and a convex calyx (CV). The metabolomic characteristics of the Korla fragrant pear fruits with these three different appearances were assessed by multivariate statistical analysis, including principal component analysis (PCA) and orthogonal partial least squares discriminant analysis (OPLS-DA), to understand the mechanisms of pear fruit quality variation. Moreover, the DPPH free radical-scavenging and ABTS cation radical-scavenging capacities were investigated to assess the antioxidant capacity of these fruits with different appearances. The results obtained not only provide information on the differentially accumulated metabolites (DAMs) affecting the quality of Korla fragrant pear fruits but also promote an understanding of how to improve the quality of Korla fragrant pear fruits and the grading, processing, and use of poor-quality fruits.

## 2. Results

### 2.1. Morphological Differences among Fruits with a Convex Calyx and Rough Skin (RS), Calyx Shedding (SD), and a Convex Calyx (CV)

**Figure 1:**
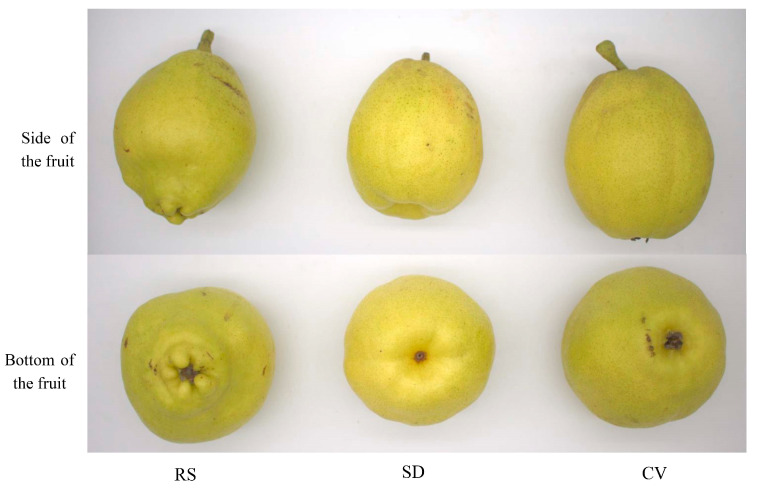
Morphologies of fruits with a convex calyx and rough skin, calyx shedding, and a convex calyx.

RS and CV fruits are phenotypically indistinguishable during early fruit development, and changes generally begin to appear later in fruit development. Therefore, RS, SD, and CV fruits were selected at the end of August for metabolomics analysis in this study. As shown in Figure 1, the RS fruits had a persistent calyx with a less beautiful appearance, a more irregular fruit shape, and rougher skin compared with the SD fruits.

### 2.2. Variations in the Main Flavor Substances in Fruits with Different Appearances

**Figure 2:**
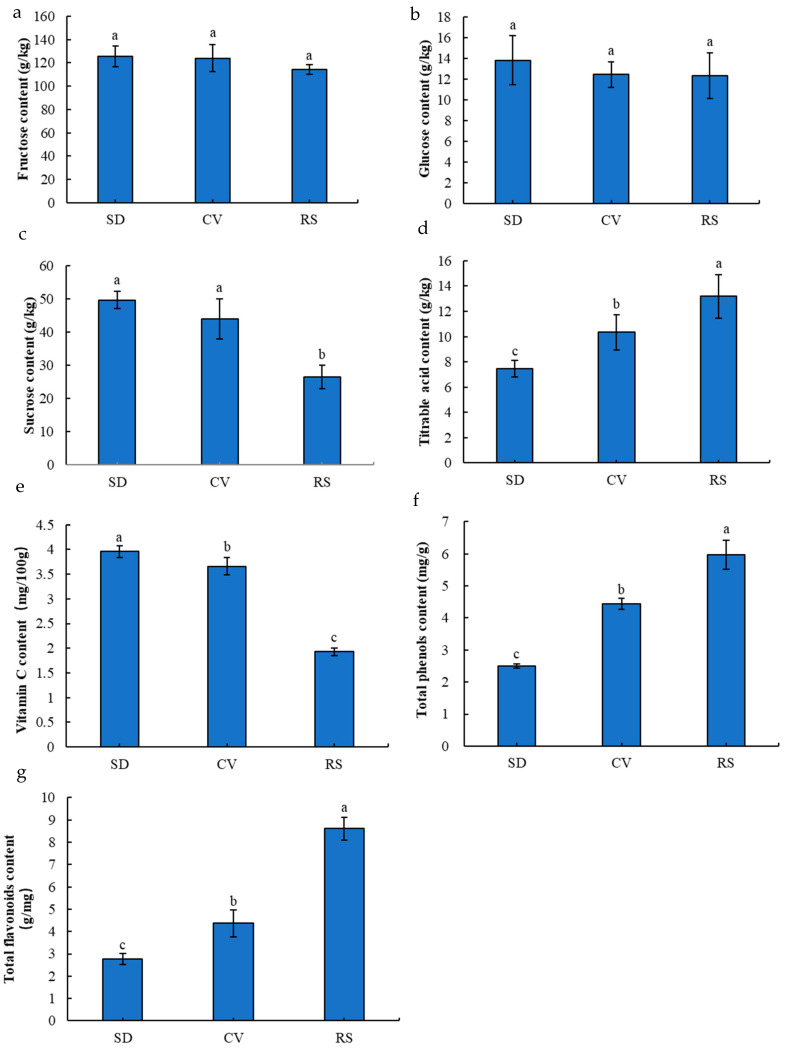
Sugar composition, titratable acids, vitamin C, and major secondary metabolites in the pulp of fruits with different appearances. (**a**–**d**) Contents of fructose, glucose, sucrose, and titratable acid in the pulps of the fruits. (**e**–**g**) Contents of vitamin C, total phenols, and total flavonoids in the fruit pulps. The error bars represent the means ± SDs (*n* = 3), and different lowercase letters indicate significant differences (*p* < 0.05) between fruits with different appearances.

Fruit flavor is one of the most important factors affecting the organoleptic quality of a product. Therefore, in this study, the contents of sugars (fructose, glucose, and sucrose), titratable acids, vitamin C, and secondary metabolites (total phenols and total flavonoids) were analyzed to identify metabolites associated with changes in fruit flavor (Table S1). The results showed that the fructose and glucose contents of SD fruits were not significantly different compared with CV and RS fruits, but their sucrose contents were significantly higher and increased by 12.98% and 87.54% compared with CV and RS fruits, respectively (Figure 2a–c). The titratable acid content of RS fruits was significantly higher by 76.89% and 27.57% compared with those of SD and CV fruits, respectively (Figure 2d). The vitamin C content in SD fruits was significantly higher than that in CV and RS fruits, whereas the content of secondary metabolites (total phenols and total flavonoids) was significantly higher in RS fruits than in SD and CV fruits (Figure 2e–g). Phenols and flavonoids are important antioxidant substances in different fruits, and a higher content of these metabolites reflects a stronger antioxidant ability [23]. These results indicate that RS fruits have a poorer flavor and decreased quality but better antioxidant activity.

### 2.3. Metabolic Profiling

Figure S1 shows the total ion current (TIC) quality control sample mass spectra obtained by UPLC–MS/MS in the positive and negative ion modes. The spectra show a high degree of overlap, demonstrating that the assay has good signal stability and reliability.

**Figure 3:**
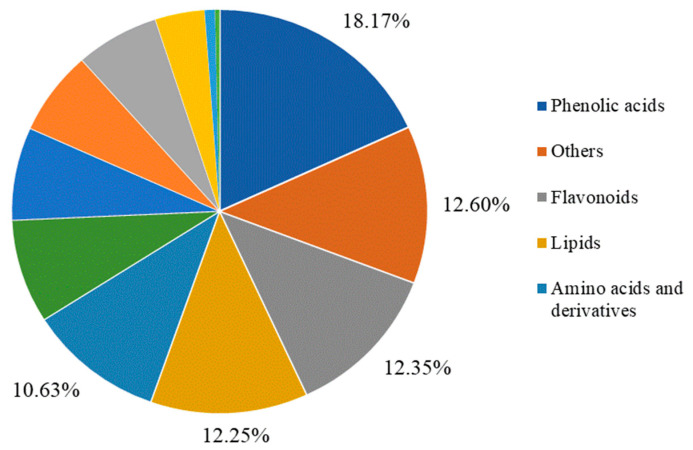
Composition analysis of the identified metabolites. The top five classes of metabolites (phenolic acids, others, flavonoids, lipids, and amino acids and derivatives) are shown next to the chart. The last seven classes are alkaloids, terpenoids, lignans and coumarins, organic acids, nucleotides and derivatives, quinones, tannins.

The metabolites in the pear fruit pulps from the CV, SD, and RS groups were studied on the basis of UPLC–ESI–MS/MS and databases. A total of 1976 metabolites were detected and identified in the pulps of the fragrant pear fruits with three different appearances. The metabolites were classified into 12 categories, including 359 phenolic acids, 249 others, 244 flavonoids, 242 lipids, 210 amino acids and derivatives, 164 alkaloids, 147 terpenoids, 133 lignans and coumarins, 129 organic acids, 76 nucleotides and derivatives, 16 quinones, and seven tannins (Table S2). Among them, phenolic acids were the most abundant with 18.17% of the total metabolites; others and flavonoids were the next most abundant with 12.60% and 12.35%, respectively; and tannins were the least abundant with 0.35% (Figure 3). These results indicated that the metabolite composition of the pulps from fragrant pear fruits with different appearances varied significantly.

### 2.4. Multivariate Statistical Analysis of the Metabolites

**Figure 4:**
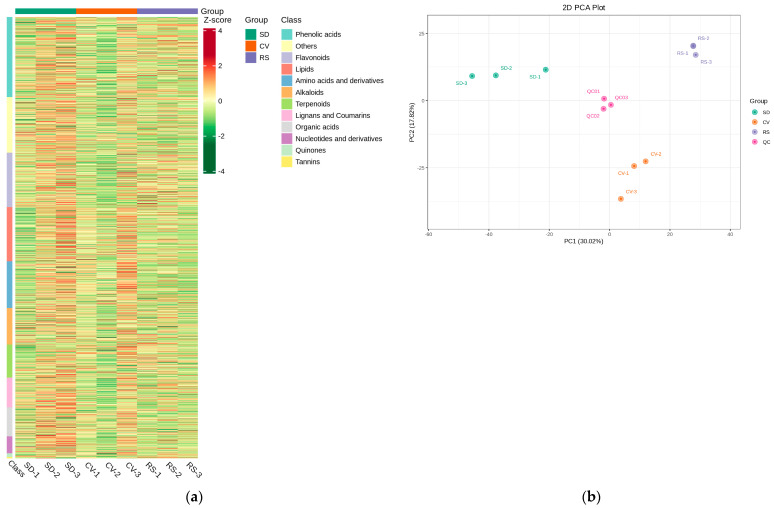
Multivariate statistical analysis of the identified metabolites. (**a**) Clustering heatmap of the identified metabolites. The sample name is listed horizontally, the metabolite information is listed vertically, Group indicates the grouping, and Class indicates the first class of the substance. Different colors are used based on the values obtained after standardization of the relative contents, and the shades reveal the content level, where red represents high content and green represents low content. (**b**) 2D PCA score plots from the mass spectrometry data of the samples in each group and the quality control samples.

**Figure 5:**
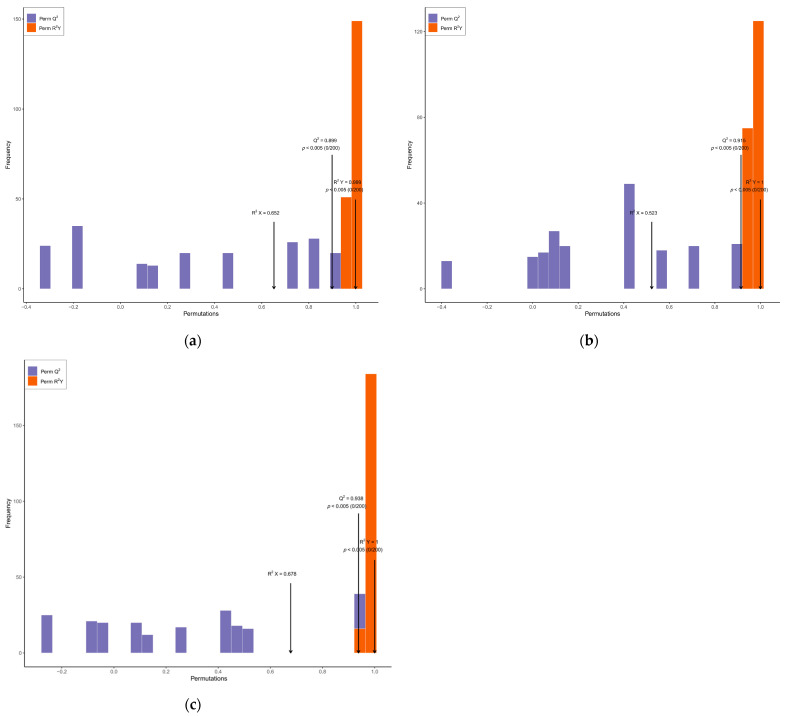
Differential metabolite pairwise comparison OPLS–DA model plots. (**a**) OPLS–DA model plot for CV vs. SD. (**b**) OPLS–DA model plot for RS vs. CV. (**c**) OPLS–DA model plot for RS vs. SD.

In this study, the patterns of metabolite accumulation among fruits with different appearances were analyzed by hierarchical clustering, which indicated their differences in expression levels. Most of the phenolic acids, others, flavonoids, and lipids were increased in SD compared with CV and RS. The contents of amino acids and derivatives, alkaloids, terpenoids, lignans, and coumarins were higher in SD than in CV. However, the contents of most organic acids, nucleotides and derivatives, quinones, and tannins were decreased in RS. These data indicated that there was a clear distinction between the SD, CV, and RS fragrant pear fruits (Figure 4a). The overall metabolic differences between the CV, SD, and RS groups and the degree of variability between the samples within each group were discerned by performing PCA on the samples (including the quality control (QC) samples). In Figure 4b, the contributions of PC1 and PC2 were found to be 30.02% and 17.82%, respectively, and the significant difference between each pulp type had a cumulative contribution of 47.84%, which indicated that these two principal components could essentially reflect the main characteristics of the tested samples. In addition, the QC samples were located in the center of the PCA score plot, from which each of the three groups of samples was separated, with tight clustering of the samples within the same group. These data suggested that the results after the data processing of each sample were credible and that there were significant differences between the samples. Similarly, SD differed significantly from the other two fruit appearances, suggesting that the metabolite profile of SD was distinguishable from that of the other two fruits with different appearances and that the metabolite composition in each of the three fruits was highly distinct (Figure S2). Pairwise comparisons of pulp samples were performed using the OPLS-DA model to assess differences between CV and SD (R^2^X = 0.652, R^2^Y = 0.999, Q^2^ = 0.899; Figure 5a), RS and CV (R^2^X = 0.523, R^2^Y = 1, Q^2^ = 0.915; Figure 5b), and RS and SD (R^2^X = 0.678, R^2^Y = 1, Q^2^ = 0.938; Figure 5c). The OPLS-DA score plot showed clear separation of the metabolites from fruits with different appearances, highlighting the great differences between the metabolic profiles of the pulp samples. Moreover, the Q^2^ values of all comparison groups were close to 1, indicating that the model is stable and reliable and that differential metabolites can be screened based on VIP values.

### 2.5. Differential Metabolite Screening and Identification

**Figure 6:**
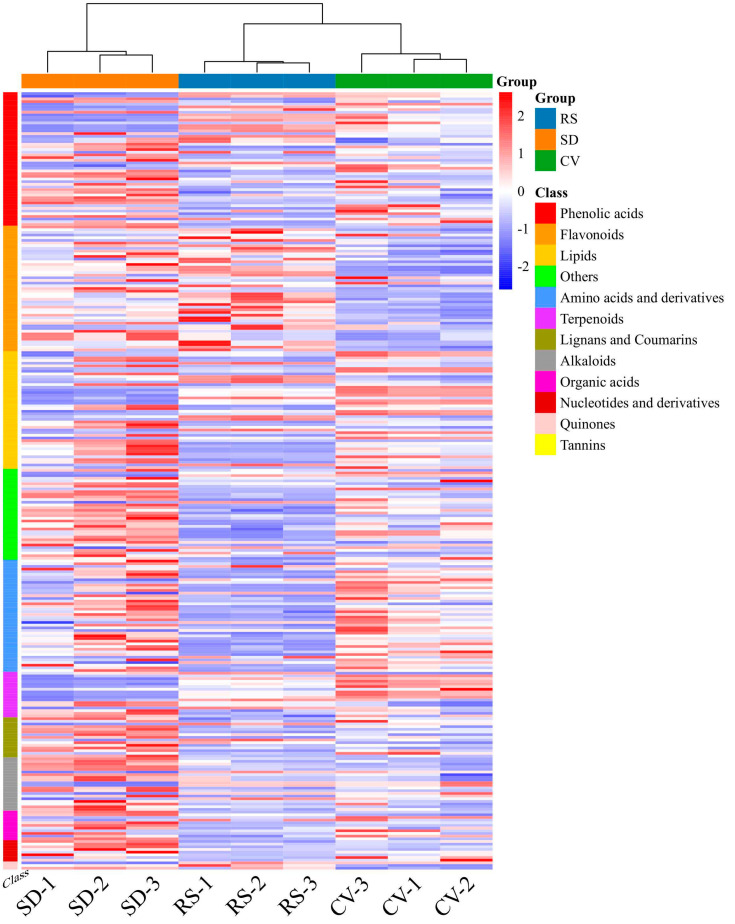
Clustering heatmap of the DAMs among RS, SD, and CV. The sample name is listed horizontally, the metabolite information is listed vertically, Group indicates the grouping, and Class indicates the first class of the substance. Different colors are used based on the values obtained after standardization of the relative contents, and the shades reveal the content level, where red represents high content and blue represents low content. Tannins are not shown in the figure due to their low number (only 4).

**Figure 7:**
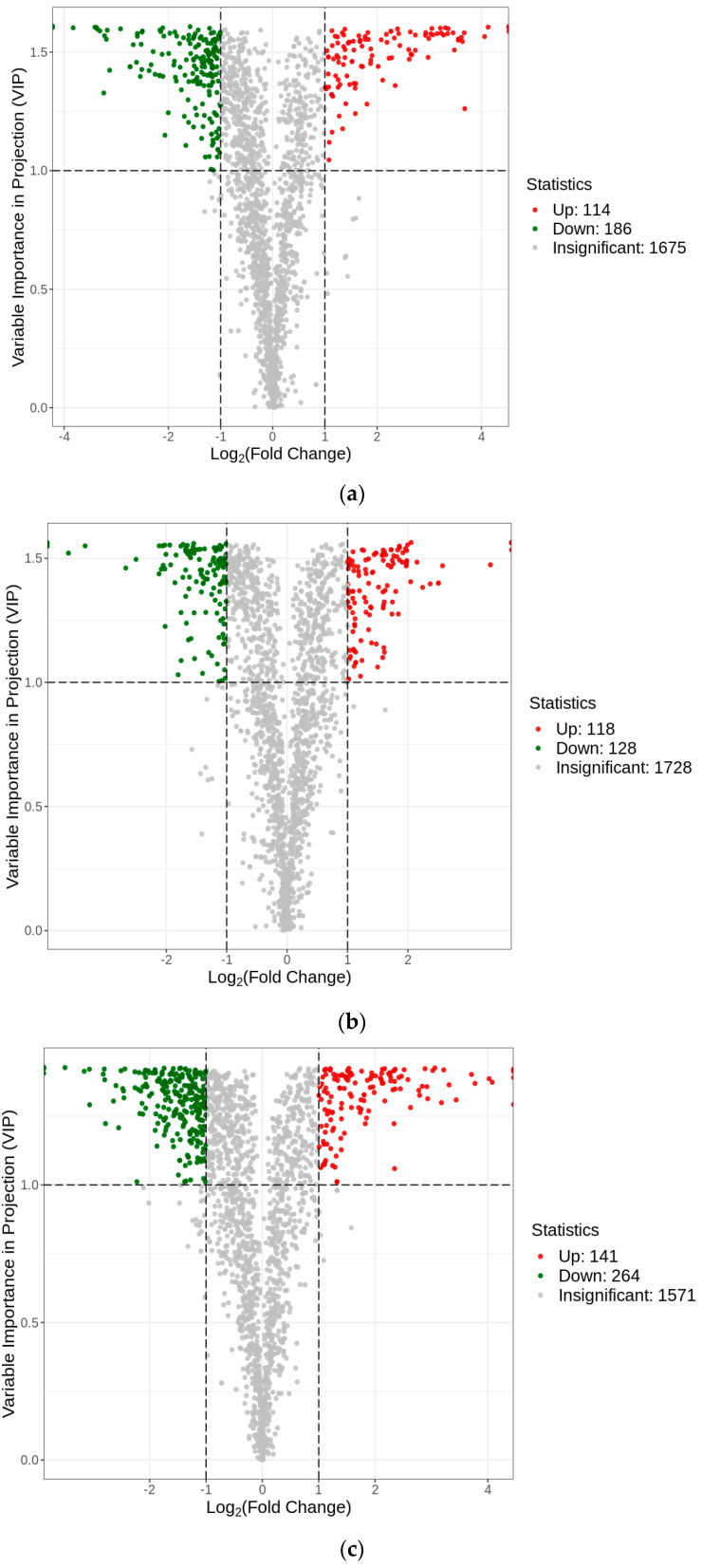
Volcano diagrams of the DAMs. (**a**) CV vs. SD; (**b**) RS vs. CV; and (**c**) RS vs. SD. Each point represents a metabolite, and the horizontal axis represents the log of the fold change of that metabolite between the two groups of samples (log2-fold change).

**Figure 8:**
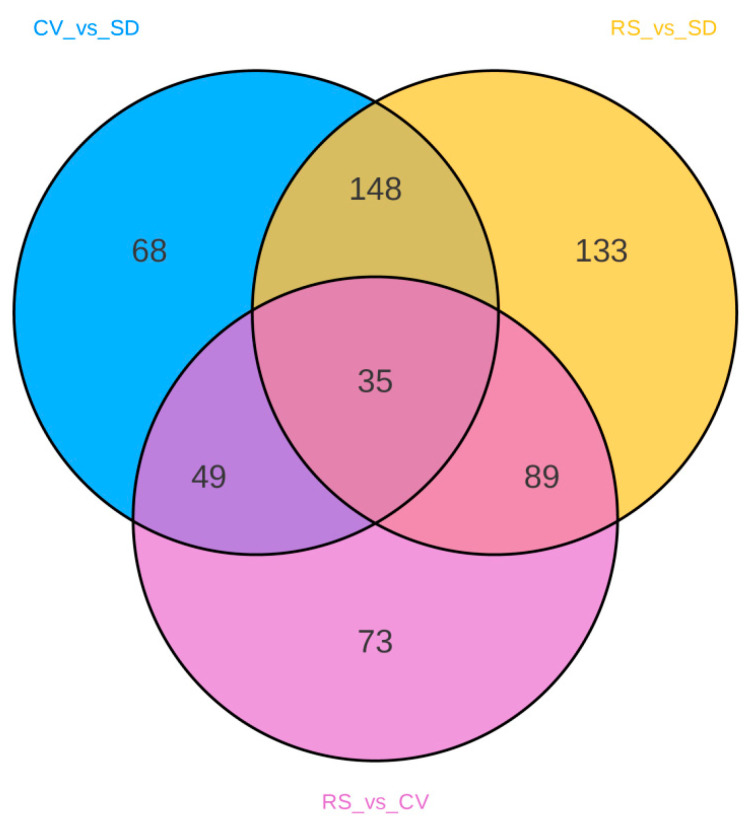
Venn diagrams of the DAMs in each comparison group from the pulp samples of three pear fruits.

Based on the OPLS-DA results, VIP values > 1, fold change ≥ 2, or fold change ≤ 0.5 were used as criteria to screen the different comparison groups for DAMs (Table S3). A total of 595 DAMs were detected in this study, and the expression levels of these DAMs in the CV and RS groups were significantly different from those in the SD group (Table S4). The results showed that more than half of the DAMs were expressed at high levels in SD and at low levels in CV and RS. The total number of metabolites with a high relative content in SD was the largest, especially phenolic acids, lipids, amino acids and derivatives, alkaloids, and organic acids. Compared with CV, RS had a more active flavonoid metabolism and more flavonoids were present, while the total number of metabolites with a high content was the lowest in CV and the accumulation of flavonoids was lower (Figure 6). The differences in metabolite expression levels in the comparison groups, as well as the statistical significance of the differences, can be clearly observed by volcano plots (Figure 7a–c). There were 300 DAMs between CV and SD (186 downregulated and 114 upregulated), 246 DAMs between RS and CV (128 downregulated and 118 upregulated), and 405 DAMs between RS and SD (264 downregulated and 141 upregulated). Screening of the DAMs showed the greatest differences between SD and the fruits with the other two appearances, followed by RS and CV. These DAMs were classified into 12 different categories, including 114 phenolic acids, 108 flavonoids, 81 lipids, 70 others, 58 amino acids and derivatives, 46 terpenoids, 40 lignans and coumarins, 30 alkaloids, 23 organic acids, 16 nucleotides and derivatives, five quinones, and four tannins. These data show that phenolic acids and flavonoids are the main metabolites. Detailed information on some of the DAMs is listed in Table 1. Furthermore, Venn diagrams were generated from the data from these three pairwise comparisons. As shown in Figure 8, each comparison group had its own specific DAMs. However, there were 35 common DAMs, including 2-hydroxycinnamic acid, chlorogenic acid (3-O-caffeoylquinic acid), naringenin-7-O-rutinoside (narirutin), hesperetin-7-O-rutinoside (hesperidin), LysoPC 12:0, maltotriose, tianshic acid, L-homomethionine, etc. (Table S5).

### 2.6. K-Means Analysis of the DAMs

**Figure 9:**
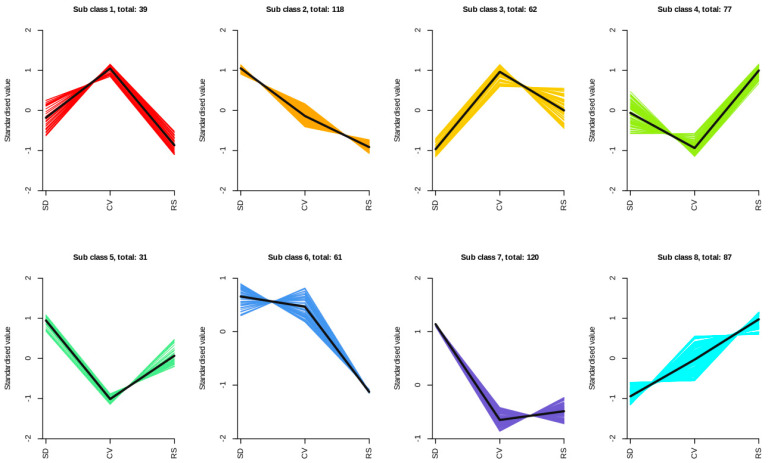
K-means diagrams of the DAMs. Horizontal coordinates correspond to SD, CV, and RS; vertical coordinates indicate the normalized metabolite relative content; subclass represents the metabolite class number with the same trend.

K-means cluster analysis is a commonly used, unsupervised analytical method for grouping samples or metabolites based on their characteristics. To investigate the change trends in the relative contents of metabolites in the fruits with different appearances, the relative contents of all DAMs identified according to the screening criteria in all comparison groups were subjected to unit variance scaling followed by K-means cluster analysis (Figure 9 and Table S6). The annotated DAMs were categorized into eight groups based on their accumulation pattern. Subclass 8 contained 87 metabolites, whose contents in fragrant pear pulp increased as the pulp shape changed, reaching the highest level in RS. This indicates that the DAMs in Subclass 8 are key metabolites in RS pulp. Representative metabolites in this subclass include sinapic acid, 3,4-digalloylshikimic acid, quercetin-7-O-glucoside, naringenin-4′-O-glucoside, dihydrokaempferol-7-O-glucoside, 17-hydroxylinolenic acid, L-asparagine, and L-ascorbic acid (vitamin C), and they were all significantly upregulated in RS vs. SD. Subclasses 2 and 6 consist of 179 metabolites whose contents in the pulp of fragrant pears decreased with changes in the pulp form. Moreover, some DAMs were particularly enriched in the SD (Subclasses 5 and 7), CV (Subclasses 1 and 3), and RS (Subclass 4) groups. Notably, the metabolites in Subclass 8 perfectly reflect the metabolites that are predominantly upregulated.

### 2.7. KEGG Enrichment Analysis of the DAMs

**Figure 10:**
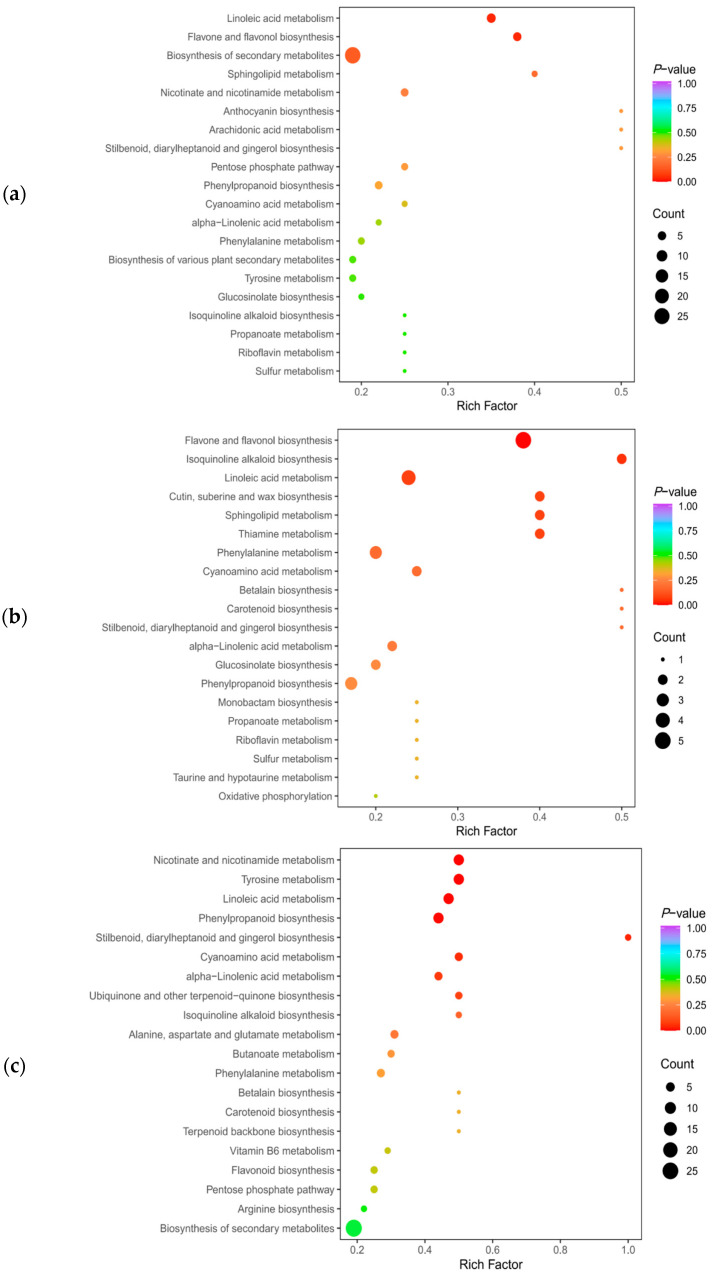
KEGG pathway analysis of the DAMs in CV vs. SD (**a**), RS vs. CV (**b**), and RS vs. SD (**c**). The horizontal axis indicates the corresponding rich factor for each pathway, and the vertical axis gives the name of the pathway (sorted by *p* value). The color of the dots reflects the size of the *p* value, with redder indicating more significant enrichment. The size of the dot indicates the number of enriched DAMs.

Metabolic pathway enrichment analysis of the screened DAMs was performed with the KEGG platform to understand their change mechanisms in fruits with different appearances. DAMs were annotated with 51 metabolic pathways in CV vs. SD. As shown in Figure 10a, the DAMs were mainly distributed in 20 metabolic pathways, including linoleic acid metabolism, flavone and flavonol biosynthesis, the biosynthesis of secondary metabolites, and sphingolipid metabolism. Among them, two metabolic pathways, linoleic acid metabolism and flavone and flavonol biosynthesis, were significantly enriched (*p* < 0.05), with six and five DAMs, respectively. In RS vs. CV, the DAMs were annotated to 41 metabolic pathways, such as flavone and flavonol biosynthesis, isoquinoline alkaloid biosynthesis, linoleic acid metabolism, and cutin, suberin, and wax biosynthesis. Among them, only the flavone and flavonol biosynthesis metabolic pathways were significantly enriched (*p* < 0.05) (Figure 10b). There were five DAMs involved in the flavone and flavonol biosynthesis pathways, including quercetin-3-*O*-rutinoside (rutin), quercetin-3-*O*-glucoside (isoquercitrin), quercetin-3-*O*-sambubioside, kaempferol-3-*O*-galactoside (trifolin), and kaempferol-3-*O*-rutinoside (nicotiflorin), and these five DAMs were metabolized more actively and were present in higher contents in RS. The DAMs between RS and SD were annotated to 56 metabolic pathways and were significantly enriched (*p* < 0.05) in six metabolic pathways, namely, nicotinate and nicotinamide metabolism, tyrosine metabolism, linoleic acid metabolism, phenylpropanoid biosynthesis, stilbenoid, diarylheptanoid, and gingerol biosynthesis, and cyanoamino acid metabolism, where there were eight, eight, eight, eight, two, and four DAMs, respectively (Figure 10c). The results showed that these DAMs were mainly enriched in the metabolic pathways of flavone and flavonol biosynthesis, linoleic acid metabolism, and phenylpropanoid biosynthesis, and that the accumulation of different DAMs in these metabolic pathways might have caused the differences in fruit appearance.

### 2.8. Comparison of Antioxidant Activities among Fruits with Different Appearances

**Figure 11:**
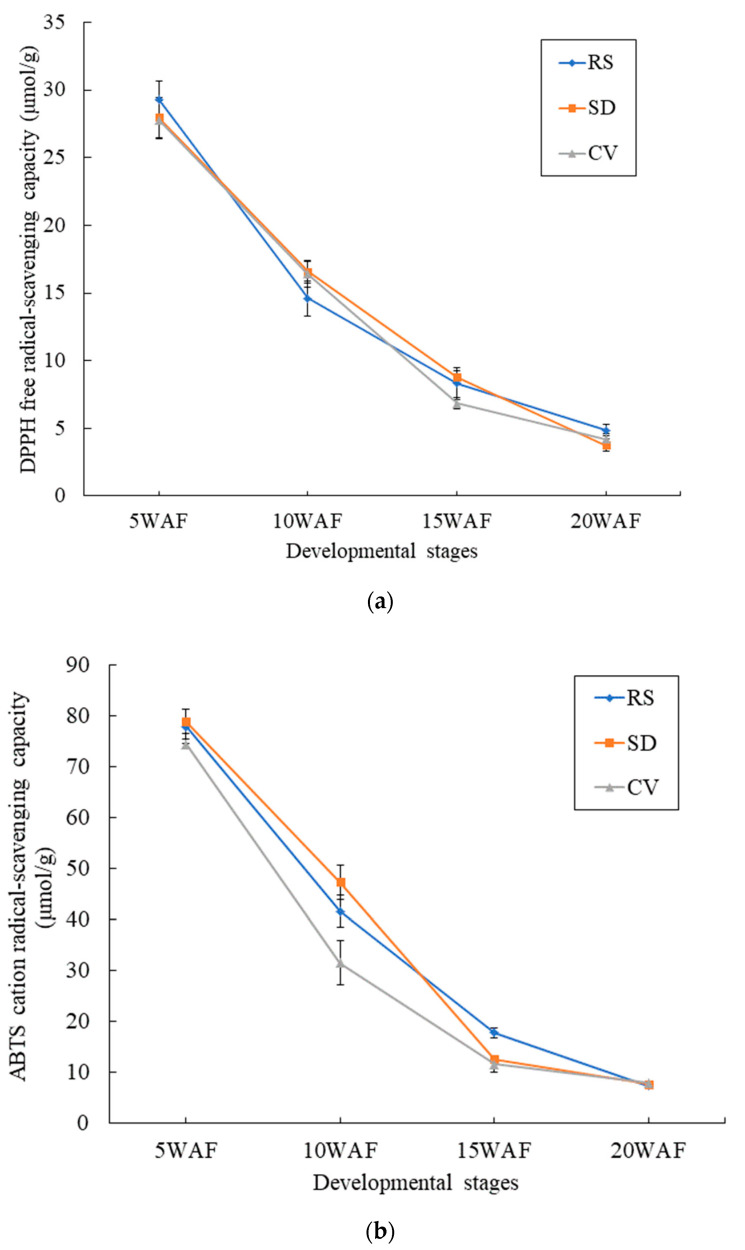
Comparison of the antioxidant activity among fruits with different appearances at different developmental stages. (**a**) DPPH-free radical-scavenging capacity. (**b**) ABTS cation radical-scavenging capacity.

As shown in Figure 11, the DPPH free radical-scavenging and ABTS cation radical-scavenging capacities of the pulp of Korla fragrant pear fruits showed a decreasing trend during growth and development. RS fruits exhibited the strongest DPPH free radical-scavenging capacity of 29.32 μmol/g at 5 weeks after flower blooming (WAF), which was slightly higher than that of SD and CV fruits. At 15 WAF, RS fruits had a higher DPPH free radical-scavenging capacity than CV fruits. The DPPH free radical-scavenging capacity did not show clear differences among the three fruits at 20 WAF, but the RS fruits had a slightly higher capacity than the SD and CV fruits. The ABTS cation radical-scavenging capacity is an important indicator of the antioxidant effect of a reactive substance. At 5 WAF and 10 WAF, the ABTS cation radical-scavenging capacity of SD fruits was slightly higher than that of RS and CV fruits. At 15 WAF, the ABTS cation radical-scavenging capacity of RS fruits reached 17.75 μmol/g, whereas those of SD and CV fruits reached 12.60 μmol/g and 11.56 μmol/g, respectively. The ABTS cation radical-scavenging capacity at 20 WAF did not show an obvious difference between the three fruits (Table S7). The results showed that the antioxidant activity of RS fruits became stronger during the development and maturity of the fruit, and the extract of this fruit could be used in food processing and other fields.

## 3. Discussion

Metabolomics can allow the identification and quantification of all metabolites in a specific tissue or organism that can be considered biomarkers for identifying fruit quality-related factors [24,25]. Among the three metabolomics sequencing technologies, widely targeted metabolomics integrates the advantages of both targeted and nontargeted metabolomics [26]. The UPLC-MS/MS-based approach to widely targeted metabolomics is becoming increasingly popular for analyzing and characterizing plant metabolites as a rapid and reliable methodology [27]. Korla fragrant pear fruits contain a variety of mineral elements and are widely used in jam processing, fruit wine distillation, and functional substance extraction [28]. In addition, fragrant pear fruits have high medicinal value due to their free radical scavenging, anti-inflammatory, analgesic, and antipyretic effects [29]. In Korla fragrant pears, the calyxes of some flowers are deciduous while others are persistent, and persistent calyxes are the main cause of deformed fruits among these pears, which negatively affects their shape and quality and directly affects their economic efficiency [7]. Therefore, we used nutrient composition analysis and UPLC-MS/MS-based widely targeted metabolomics approaches to analyze the changes in the quality characteristics of Korla fragrant pear fruits with a convex calyx and rough skin, calyx shedding, and a convex calyx. Moreover, the DPPH free radical-scavenging and ABTS cation radical-scavenging capacities were used to assess the antioxidant capacity of the fruits with different appearances. The nutrient composition analysis showed that the fructose, glucose, sucrose, and vitamin C contents were significantly higher in SD fruits than in CV and RS fruits. However, high levels of flavor substances such as titratable acids, total phenols, and total flavonoids were found in the pulp of RS fruits. A metabolomics approach was used to measure 1976 metabolites in the pulp of Korla fragrant pears, providing a broader scale with which fragrant pear metabolites could be investigated. These data help to gain insight into the metabolomic landscape of fragrant pears, comprehensively analyze changes in the metabolome during fruit development, and provide a foundation to understand the metabolic basis of the important quality traits in commercial fragrant pears.

A total of 595 DAMs were screened by FC and VIP values. These DAMs best represent the differences between the pulp of the Korla fragrant pear fruits with different appearances, which can be used as references for future research. Analysis of the DAMs between CV, SD, and RS showed that phenolic acids and flavonoids were the main metabolites. Phenolic compounds are mainly categorized as phenolic acids, flavonoids, tannins, lignans, and coumarins, which are the most widely distributed secondary metabolites during the ripening stage of fruits and vegetables [30,31]. Phenolic acids are closely related to sensory indicators such as fruit color, flavor, and texture and have good nutritional functions and pharmacological effects such as antioxidant activity [32,33]. In the present study, 114 phenolic acids were screened, and their contents accumulated differently in the Korla fragrant pear fruits with different appearances, with most of the phenolic acids in SD being present at significantly higher levels than those in CV and RS (Figure S3). Among them, chlorogenic acid (3-*O*-caffeoylquinic acid), 1-*O*-sinapoyl-β-D-glucose, and sinapyl alcohol, which are metabolites involved in phenylpropanoid biosynthesis, were significantly upregulated in SD. It has been shown that the phenylpropanoid biosynthesis pathway begins with phenylalanine, and the flow of metabolites from this pathway reduces lignin synthesis [34], which may improve SD fruit palatability. Chlorogenic acid substances are very important functional components of medicinal plants, as they possess various bioactivities such as antioxidant, anti-inflammatory, bacteriostatic, and immunomodulatory activities [35]. Chlorogenic acids, the major phenolic acids produced by secondary metabolism in many plants, have been reported to be abundant in Korla fragrant pear fruits [36]. The abundance of phenolic acids may give SD fruits broad prospects for the utilization of these fruits in health applications.

**Figure 12:**
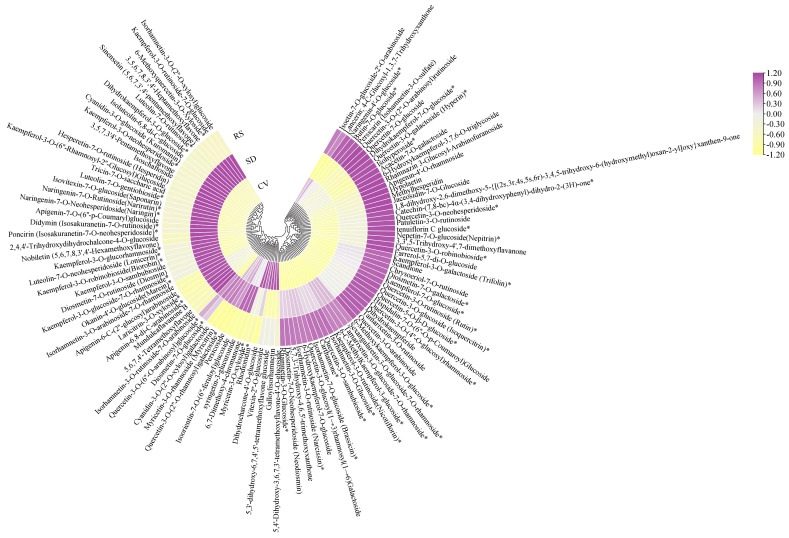
Clustering heatmap of 108 flavonoids among RS, SD and CV. The color shade reveals the content level, where purple represents high content and yellow represents low content. The asterisks represent an isomer.

Flavonoids play important roles in plant biological processes, including coloration, cell wall formation, and defense against diseases, which are beneficial to human health [37,38]. From a structural perspective, flavonoids can be classified as flavones, flavonols, flavanols, anthocyanins, and isoflavones [39]. Flavonoids have been found to scavenge reactive oxygen species in several ways, such as by scavenging free radicals and enhancing antioxidant properties [8]. Li et al. [40] analyzed the content and antioxidant capacity of total phenols and total flavonoids in eight varieties of pears in China and found that those with a high content of total phenols and total flavonoids had significantly higher antioxidant capacities. The nutrient composition analysis conducted in this study showed that flavor substances such as total phenols and total flavonoids were more abundant in the pulp of RS fruits than in the pulp of SD and CV fruits. The DPPH free radical-scavenging and ABTS cation radical-scavenging capacities are important indicators of the antioxidant effects of reactive substances. The results of this study showed that during the development and maturation of the fruits, the RS fruits had stronger antioxidant activity than SD and CV fruits. The results indicate that RS fruits have better antioxidant activity and that their extracts can be used in the fields of medicine and functional food processing. Furthermore, in the present study, 108 flavonoids were screened, and their contents accumulated differently among the fruits with three different appearances. Among them, RS had a more active flavonoid metabolism, and flavonoids were more abundant than in SD and CV, including quercetin-3-*O*-rutinoside (rutin), quercetin-3-*O*-glucoside (isoquercitrin), quercetin-3-*O*-sambubioside, kaempferol-3-*O*-galactoside (trifolin), and kaempferol-3-*O*-rutinoside (Nicotiflorin). These flavonoid compounds are metabolites involved in flavone and flavonol biosynthesis and are abundant in Korla fragrant pear RS fruits (Figure 12). KEGG enrichment analysis showed that the DAMs were mainly enriched in metabolic pathways such as flavone and flavonol biosynthesis and phenylpropanoid biosynthesis. Both flavones and flavonols are synthesized through the phenylpropanoid pathway, and phenylpropanoids are key components in lignin biosynthesis and one of the most important determinants of fruit quality [41]. Lignin has been reported to be the main component of pear stone cells, and the average lignin content of pear stone cells is higher than the cellulose content [42]. The lignified stone cell content is a key factor when evaluating the quality of pear fruit, as it affects the economic value of the fruit [43]. Some pear fruits have coarse and slaggy textures due to their high stone cell contents, which seriously affects their edible quality [44]. Moreover, flavonoids are the main source of bitter flavors in fruits and are natural antioxidants that contribute to human health [45]. Flavanols form complex polymers in fruits, which have a bitter and rough taste, and their effect on the flavor of fruit changes during ripening due to polymerization and hydrolysis [46]. In this article, it was determined that catechin-(7,8-bc)-4α-(3,4-dihydroxyphenyl)-dihydro-2-(3H)-one is the most abundant flavanol in RS. In summary, we speculated that this is the reason why the pulp of Korla fragrant pear fruits with a rough skin is harder, less juicy, more slaggy, and less flavorful and can be widely used in the processing of antioxidant functional foods such as fruit juices, jams, and fruit wines [5].

**Figure 13:**
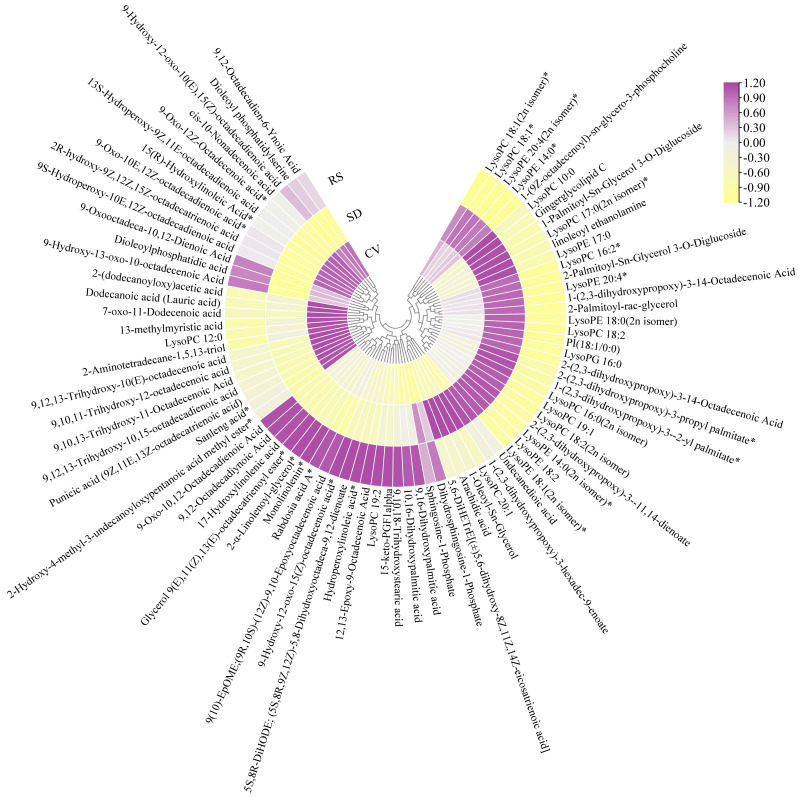
Clustering heatmap of 81 lipids among RS, SD, and CV. The color shade reveals the content level, where purple represents high content and yellow represents low content. The asterisks represent an isomer.

Lipids and amino acids have been reported to be key factors in fruit quality [47]. Lipids are precursors of volatiles that contribute to fruit odor, while the levels of volatiles affect fruit palatability and flavor [48]. In this study, 81 lipids were screened, most of which were present at significantly higher levels in SD than in CV and RS (Figure 13), including naturally occurring cyclic bioactive lipid molecules such as LysoPC 18:1, LysoPC 18:2, LysoPC 19:1, and LysoPC 20:1 [49], which play key roles in the flavor and taste of fruits. Amino acids are associated with fruit quality traits, not only as precursors of volatile compounds in aroma but also as contributors to the taste of the pear fruit [50,51]. Of the 58 amino acids and derivatives screened in this study, most of the differential amino acids and derivatives in SD were significantly higher than those in CV and RS (Figure S4), including flavor-presenting amino acids such as glycine, alanine, serine, aspartic acid, etc. Moreover, glycine, alanine, and aspartic acid contribute to the sweetness of the pear and play a role in the formation of the flavor of the Korla fragrant pear [52]. Phenylalanine and tyrosine are aromatic amino acids that are involved in phenylalanine metabolism in plants and are precursors for the synthesis of phenolic acids, flavonoids, and lignin, which are closely related to fruit growth, development, and quality [53]. It can be speculated that the unique, strong flavor and aroma of SD fruits are related to the abundance of polyphenols, lipids, and amino acids in the fruit.

Sugars are the major soluble constituents in ripe fruits and play a crucial role in fruit taste and flavor [54]. It has been established that sugars mainly contribute to the sweetness of fruits, and their content and composition are important indicators for determining the quality advantages and disadvantages of fruit [55,56]. Vitamin C is an important antioxidant in many fruits, and its content is a major indicator of fruit quality, as vitamin C can be involved in growth and development processes such as plant cell division and cell expansion [57,58]. Previous studies have shown that Korla fragrant pear fruits with calyx shedding had higher titers of soluble sugars and vitamin C and lower titers of titratable acids than those with persistent calyxes [14], and this finding was consistent with the results from the nutrient composition analysis conducted in the present study. Moreover, in this paper, 70 DAMs in “other” categories, including 23 sugars and 10 vitamins, were screened from the pulp of the Korla fragrant pear fruits with three different appearances (Table S4). The levels of twenty-one sugars, including D-sucrose, D-maltose, D-lactose, planteose, and nystose, were significantly higher in SD than in RS, and the levels of seven vitamins, such as riboflavin (vitamin B2), nicotinic acid (vitamin B3), and isoascorbic acid 2-O-glucoside, were significantly higher in RS than in SD. In RS, only two sugars (manninotriose and D-erythrose-4-phosphate) and two vitamins (L-ascorbic acid (vitamin C) and N-(beta-D-glucosyl)nicotinate)) were present at higher levels than in SD and CV, whereas the level of only one vitamin (pyridoxal-5′-phosphate) was significantly higher in CV than in SD and RS (Figure S5). This indicates that sweetness was the most pronounced in SD fruits, followed by CV, and the flavor quality was better than that in RS. Sugars such as sucrose, lactose, and maltose have been reported to be the basic substances needed for plant life activities, representing metabolic resources for carbon skeleton structures and acting as an energy supply to promote plant growth and development, as well as an important contributor to the sweetness and flavor of many fleshy fruits [59].

Organic acids are important indicators of the taste and nutritional quality of appetizing fruits with digestive properties [60]. In this study, twenty-three different organic acids were screened, among which seventeen (3-methyl-shikimic acid, 2-propylsuccinic acid, and azelaic acid, etc.) were found in significantly higher contents in SD than in CV and RS, four (tianshic acid, etc.) were found in higher contents in CV than in SD and RS, and only two (fumaric acid and DL-glyceraldehyde-3-phosphate) were found in significantly higher contents in RS than in SD and CV (Figure S6). This suggested that abundant organic acids accumulate in SD to enrich the flavor of SD fruits. Studies have also shown that organic acids are not only important sources of fruit attributes such as acidity and antioxidant molecules but also play key roles as signaling molecules to regulate plant development, stress tolerance, and yield [61]. Moreover, fruits contain a variety of functional substances that are beneficial to the human body, such as alkaloids, phenols, and saponins, which have antioxidant, antimicrobial, immune-boosting, and sedative effects [62,63]. Most alkaloids have significant biological activities, and they are some of the most important active ingredients in Chinese herbal medicines. In this paper, 30 alkaloids were screened, of which 24 were found at higher levels in SD than in CV and RS, including nicotianamine, 6-acetyldelpheline, and feruloylhistamine. Only six alkaloids were found at higher levels in RS than in SD, including L-tyramine, trigonelline, and dhurrin (Figure S7). These results indicate that SD is richer in medicinally beneficial alkaloids that have outstanding biological activities [64]. We also found an interesting phenomenon in which the levels of most terpenoids in CV were significantly higher than those in RS and SD (Figure S8). Terpenoids are usually produced in nutrient tissues and occasionally in roots [65]. Terpenoids mainly include low-molecular-weight monoterpenes, sesquiterpenes, diterpenes, and triterpene derivatives, which are widely present in plant tissues such as leaves, flowers, and fruits and have anti-inflammatory effects and inhibit tumor cell proliferation [66,67]. These data show that the contents of anti-inflammatory and antitumor compounds are higher in CV than in RS and SD.

## 4. Materials and Methods

### 4.1. Plant Material

The experiment was performed in the spring of 2022 at the experimental base of the Xinjiang Academy of Agricultural Reclamation Sciences. At the end of August of that year (20 weeks after flower blooming), fruits with a convex calyx and rough skin, calyx shedding, and a convex calyx (mature, disease-free, and undamaged) were selected from the test plants. Specifically, pear fruits with a convex calyx and rough skin (RS), calyx shedding (SD), and convex calyx (CV) were collected, which yielded a total of nine sample groups labeled RS-1, RS-2, and RS-3; SD-1, SD-2, and SD-3; CV-1, CV-2, and CV-3, respectively. These fruits obtained 20 weeks after flower blooming were used for metabolomics and nutrient composition analysis. Intact fruits were packed in foam boxes and stored at 4 °C for transport to the laboratory. In the laboratory, the fragrant pear fruits were peeled, and the pulp was cut into 0.5 cm × 0.5 cm pieces. The pear pulps were immediately placed in tubes and frozen in liquid nitrogen for use in subsequent metabolomics and nutrient composition analyses. Moreover, the antioxidant activity of RS, SD, and CV fruit samples of Korla fragrant pears collected 5 weeks after flower blooming (5 WAF), 10 weeks after flower blooming (10 WAF), 15 weeks after flower blooming (15 WAF), and 20 weeks after flower blooming (20 WAF) was measured. The samples at each developmental stage were replicated three times.

### 4.2. Nutrient Compositions Analysis of Pear Fruits

In this study, the sugar compositions were analyzed according to the anthrone colorimetry method described by Mamat et al. [8]. Briefly, 0.3 g of grinded powder was boiled in 15 mL of distilled water for 30 min, and the extract was then centrifuged at 12,000× *g* for 15 min at 4 °C. Subsequently, the supernatant was placed into a 25-mL volumetric flask and diluted to scale. After filtration, the filtrate was used to determine the fructose, glucose, and sucrose contents. The titratable acid and vitamin C contents were determined by NaOH acid-base titration and molybdenum blue spectrophotometry, respectively, as described by Aubert et al. [68]. The total phenol content was determined by the Folin-phenol colorimetric method as described by Li et al. [69]. The total flavonoid content was determined as described by Mamat et al. [8].

### 4.3. Fruit Sample Preparation and Extraction

Each sample was removed from the −80 °C freezer, thawed on ice, and vortexed for 30 s to mix well. Three milliliters of the sample was taken for vacuum freeze-drying. Then, 500 μL of 70% methanol internal standard extract was added, and the sample was vortexed for 15 min and ultrasonicated for 10 min. The mixture was then centrifuged at 12,000 r/min for 3 min at 4 °C. The supernatant was removed with a pipette, and the remaining material was filtered through a microporous membrane (0.22 μm) and stored in an injection vial for LC–MS/MS detection.

### 4.4. UPLC Conditions

The sample extracts were analyzed at Wuhan MetWare Biotechnology Co., Ltd. (Wuhan, China) using a UPLC-ESI-MS/MS system (UPLC, Nexera X2, Shimadazu, Kyoto, Japan; MS, 6500 Q TRAP, Applied Biosystems, Norwalk, CT, USA). The analysis was performed according to the company’s standard procedures [70], and the metabolome data of this experiment were generated by a combination of comparison with the company’s local database and manual identification. The analytical conditions were as follows. For UPLC, the column used was an Agilent SB-C18 (1.8 µm, 2.1 mm × 100 mm), and the mobile phase consisted of solvent A: pure water (Merck, Shanghai, China) with 0.1% formic acid (Aladdin, Shanghai, China) and solvent B: acetonitrile (Merck) with 0.1% formic acid. Sample measurements were performed with gradient elution at the starting conditions of 95% A; 5% B. Within 9 min, a linear gradient to 5% A; 95% B was performed, and the composition of 5% A; 95% B was maintained for 1 min. Subsequently, the mobile phase composition was changed to 95% A, 5.0% B within 1.1 min, where it was maintained for 2.9 min. The flow rate was set at 0.35 mL per minute; the column oven was set to 40 °C; and the injection volume was 2 μL. The effluent was alternatively connected to an ESI-triple quadrupole linear ion trap (QTRAP, Norwalk, CT, USA) mass spectrometer.

### 4.5. ESI-Q TRAP-MS/MS

Linear ion trap (LIT) and triple quadrupole (QQQ) scans were acquired on a Q TRAP-MS, API 6500 Q TRAP LC/MS/MS System equipped with an ESI Turbo Ion-Spray interface, operating in a positive ion mode, and controlled by the Analyst 1.6.3 software (AB Sciex, Concord, ON, Canada). The ESI source operation parameters were as follows: source temperature 500 °C; ion spray voltage (IS) 5500 V (positive ion mode)/ −4500 V (negative ion mode); ion source gas I (GSI), gas II (GSII), and curtain gas (CUR) at 50, 60, and 25 psi, respectively; and the collision-activated dissociation (CAD) was set to high. Instrument tuning and mass calibration were performed in QQQ and LIT modes with 10 and 100 μmol/L polypropylene glycol solutions, respectively. Based on the metabolite information in the self-built MetWare Database (http://www.metware.cn/, accessed on 20 April 2022) and public databases, the materials were qualitatively analyzed according to the secondary spectrum information, and the isotope signal was removed during the analysis [70]. QQQ scans were acquired as MRM experiments with the collision gas (nitrogen) set to medium. The declustering potential (DP) and collision energy (CE) for individual MRM transitions were determined with further DP and CE optimization. A specific set of MRM transitions was monitored for each period according to the metabolites that eluted in this period.

Metabolite quantification was accomplished using MRM analysis [71]. In the MRM model, the quadruple first screens the precursor ion (parent ion) of the target substance and excludes the ions corresponding to other molecular weight substances to eliminate the interference initially; the precursor ion is induced to ionize by the collision chamber and breaks up to form many fragment ions, which are then filtered through the QQQ to select a desired characteristic fragment ion and exclude the interference of non-target ions. After acquiring the metabolite profiling data of different samples, peak area integration was performed on all the peaks of the chromatograms of the substances, and the integration was corrected for the mass spectrometry peaks of the same metabolite in the different samples.

### 4.6. Statistical Analyses and Relative Quantification

#### 4.6.1. PCA

Unsupervised PCA was performed by the statistics function prcomp within R (www.r-project.org, accessed on 20 April 2022). The data were unit variance scaled before unsupervised PCA.

#### 4.6.2. Hierarchical Cluster Analysis and Pearson Correlation Coefficients

The hierarchical cluster analysis (HCA) results of the samples and metabolites are presented as heatmaps with dendrograms, while Pearson correlation coefficients (PCCs) between samples calculated by the cor function in R are presented as only heatmaps. Both HCA and PCC were carried out by the R package ComplexHeatmap. For HCA, the normalized signal intensities of the metabolites (unit variance scaling) are presented as a color spectrum.

#### 4.6.3. Differential Metabolite Selection

For two-group analysis, differential metabolites were determined by variable importance in projection (VIP) (VIP > 1) and absolute Log2FC (|Log2FC| ≥ 1.0). VIP values were extracted from the OPLS-DA results, which also contain score plots and permutation plots, and were generated using the R package MetaboAnalystR. The data were log-transformed (log2) and mean-centered before OPLS-DA. To avoid overfitting, a permutation test (with 200 permutations) was performed. After standardization and centralization, K-means analysis of differential metabolites was performed to investigate the variation in relative contents of metabolites in different samples, according to Jiang et al. [55].

#### 4.6.4. KEGG Annotation and Enrichment Analysis

Identified metabolites were annotated using the KEGG compound database (http://www.kegg.jp/kegg/compound/, accessed on 20 April 2022), and annotated metabolites were then mapped to the KEGG pathway database (http://www.kegg.jp/kegg/pathway.html, accessed on 20 April 2022). Pathways to which significantly regulated metabolites were mapped were then fed into metabolite set enrichment analysis (MSEA), and their significance was determined by hypergeometric test *p* values.

### 4.7. Determination of Antioxidant Activity of Fruits with Different Appearances

The 1,1-diphenyl-2-picrylhydrazyl radical (DPPH, Aladdin) assay was performed as described by Li et al. [72] to assess the DPPH free radical-scavenging activity of each sample. The 2,2′-azinobis (3-ethylbenzothiazoline-6-sulfonic acid) (ABTS, Aladdin) cation radical-scavenging capacity was evaluated according to the method described by Liu et al. [73]. All the samples were analyzed in triplicate.

### 4.8. Data Analysis

Excel 2010 software was used for data processing and graphing. All samples were repeated three times, and the results are presented as means ± standard deviations (SDs). One-way analysis of variance (ANOVA) and Duncan’s test were used to identify significant differences using SPSS 17.0 (SPSS Inc., Chicago, IL, USA), and *p* < 0.05 was considered to indicate significance.

## 5. Conclusions

In summary, in this study, the metabolites in the pulp of Korla fragrant pear fruits with three different appearances were determined by nutrient composition analysis and widely targeted metabolomics technology. Moreover, the DPPH free radical-scavenging and ABTS cation radical-scavenging capacities were used to assess the antioxidant capacity of the fruits with different appearances. The nutrient composition analysis showed that fructose and glucose contents were not significantly different, and sucrose and vitamin C contents were significantly higher in SD fruits compared with CV and RS fruits. However, the abundance of flavor substances such as titratable acids, total phenols, and total flavonoids was high in the pulp of RS fruits. Metabolomics clarified that phenolic acids and flavonoids were identified as the main metabolites in these fruits. There were clear differences among the metabolites in the pulp of the Korla fragrant pear fruits with three different appearances, in which the total number of metabolites with a high relative content was the highest in SD, especially phenolic acids, lipids, amino acids and derivatives, alkaloids, and organic acids, which might explain why SD fruits have a more intense flavor and more prominent nutrition and quality. Compared with CV, RS has a more active flavonoid metabolism and a higher content of flavonoids, while the total number of high-content metabolites was the lowest in CV, which also had fewer accumulated flavonoids. Differential metabolite KEGG pathway enrichment analysis also confirmed that CV had weaker flavone and flavonol biosynthesis and less accumulation of flavonoids compared with RS, which may be the reason for the less bitter flavor of CV. However, the flavonoid content in RS fruits is very high, which may be one of the reasons why RS fruits have harder pulp and are less juicy, more slaggy, and less flavorful. Moreover, the analysis of the antioxidant activity showed that during fruit development and maturation, RS fruits had stronger antioxidant activity than SD and CV fruits. The results described here provide a theoretical basis for further studies aiming to improve the quality of Korla fragrant pear fruits and the processing of these fruits. However, the antioxidant mechanism and function of other metabolites associated with an improved quality of Korla fragrant pear fruits remain unknown and warrant further analysis.

## Figures and Tables

**Table 1:** Some of the DAMs in CV vs. SD, RS vs. CV, and RS vs. SD.

Compound Class	Index	Compounds	Fold Change		
			CV vs. SD	RS vs. CV	RS vs. SD
Phenolic acids	Zaln004057	4-caffeoylshikimic acid	-	0.38	-
	mws0178	Chlorogenic acid (3-*O*-Caffeoylquinic acid)	2.11	0.16	0.33
	HJN003	1-*O*-Sinapoyl-β-D-glucose	-	0.37	0.39
	mws0853	Sinapyl alcohol	0.44	-	-
Flavonoids	MWSHY0067	Quercetin-3-*O*-rutinoside (rutin)	0.43	3.52	-
	MWSHY0046	Quercetin-3-*O*-glucoside (Isoquercitrin)	0.45	3.94	-
	Lmjp002596	Quercetin-3-*O*-sambubioside	-	2.14	-
	mws0913	Kaempferol-3-*O*-galactoside (Trifolin)	-	2.20	-
	mws0071	Apigenin-4′-*O*-rhamnoside	-	10.30	5.08
Lipids	pmp001281	LysoPC 18:1	-	-	0.44
	pmp001273	LysoPC 18:2	-	-	0.43
	Lmhp010908	LysoPC 19:1	-	-	0.44
Others	pme0519	D-Sucrose	-	0.50	-
	Lmsn000381	D-Maltose	-	0.49	-
	Lmxn000398	D-Lactose	-	0.49	-
	mws0232	Riboflavin (Vitamin B2)	0.45	-	0.36
	pme0490	Nicotinic acid (Vitamin B3)	0.48	-	0.31
	Wasn001007	Isoascorbic acid 2-*O*-glucoside	-	-	0.44
	MWSmce486	Manninotriose	-	2.33	-
	Zmzn000079	D-erythrose-4-phosphate	2.22	-	2.99
	Hmfn000531	L-Ascorbic acid (Vitamin C)	2.20	-	2.65
	pma1751	N-(beta-D-Glucosyl)nicotinate	2.38	-	3.36
Amino acids and derivatives	Lcsp000959	Gly-Val-Ala	-	-	0.41
	MW0108103	Lys-Phe	-	-	0.42
	mws0250	L-Tyrosine	-	0.49	0.41
Terpenoids	Wbmn010746	Negundoin A	2.94	-	-
	Cmmn012461	Dehydroabietic acid	-	0.30	-
Alkaloids	mws1375	Nicotianamine	0.45	-	-
	Wbmp003594	6-acetyldelpheline	-	-	0.46
	pmp000727	Feruloylhistamine	-	-	0.41
Organic acids	ZbBn002068	3-methyl-Shikimic acid	-	-	0.45
	Lmbn002072	2-Propylsuccinic acid	0.43	-	0.37
	mws0237	Azelaic acid	0.46	-	-
	Lmgn007652	Tianshic acid	10.21	0.23	2.36
	mws0376	Fumaric acid	-	-	2.15
	pme3186	DL-Glyceraldehyde-3-phosphate	-	2.12	2.22

Fold change values ≥ 2 or ≤0.5 were considered to indicate significant differences and were used as criteria for screening DAMs. “-” indicates no significant difference.

## Data Availability

The data is contained within the manuscript and Supplementary Materials.

## Supplementary Materials

The following supporting information can be downloaded at: https://www.mdpi.com/article/10.3390/plants12233981/s1, Figure S1: Mass spectrometry total ion current overlap diagram of the quality control (QC) samples; Figure S2: 3D PCA score plots from the mass spectrometry data of the samples in each group and the quality control samples; Figure S3: Clustering heatmap of 114 phenolic acids among RS, SD, and CV; Figure S4: Clustering heatmap of 58 amino acids and derivatives among RS, SD, and CV; Figure S5: Clustering heatmap of 23 sugars and 10 vitamins among RS, SD, and CV; Figure S6: Clustering heatmap of 23 organic acids among RS, SD, and CV; Figure S7: Clustering heatmap of 30 alkaloids among RS, SD, and CV; Figure S8: Clustering heatmap of 46 terpenoids among RS, SD, and CV; Table S1: Nutritional indicators of the pulp of fruits with different appearances; Table S2: Composition analysis of 1976 identified metabolites; Table S3: DAMs were detected in the three comparison groups; Table S4: A total of 595 DAMs were detected in the three groups; Table S5: A total of 35 common DAMs in the three groups; Table S6: K-Means analysis of the DAMs; Table S7: Comparison of antioxidant activity of different-appearing fruits at different developmental stages.
