# Supplementary figures and images for: Changes in the Metabolome and Nutritional Quality of Pulp from Three Types of Korla Fragrant Pears with Different Appearances as Revealed by Widely Targeted Metabolomics
Source: Plants (Basel). 2023 Nov 27;12(23):3981. doi: 10.3390/plants12233981 (PMC10707832; doi:10.3390/plants12233981)

a

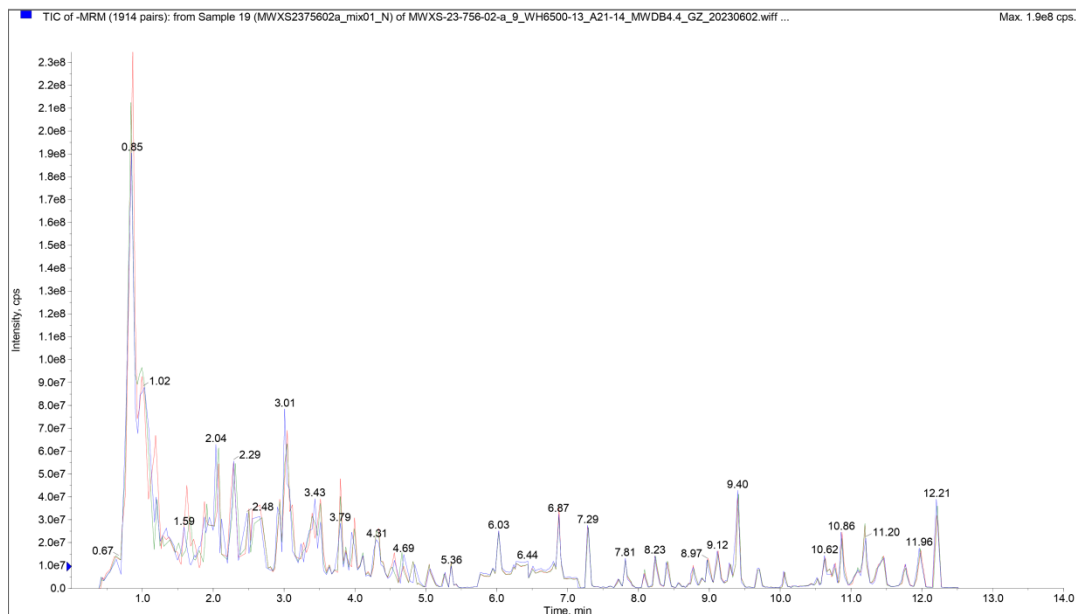

b

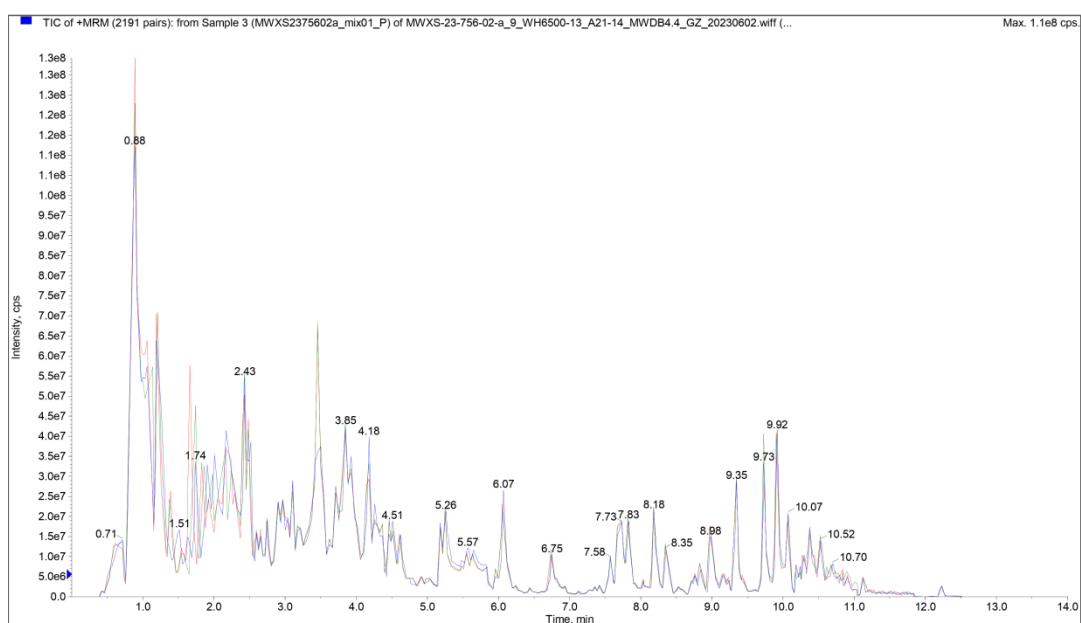

Supplement: Supplementary file 1 [file plants-12-03981-s001.zip › plants-2702487-supplementary/Figure S1. Mass spectrometry total ion current overlap diagram of the quality control (QC) samples..pdf]

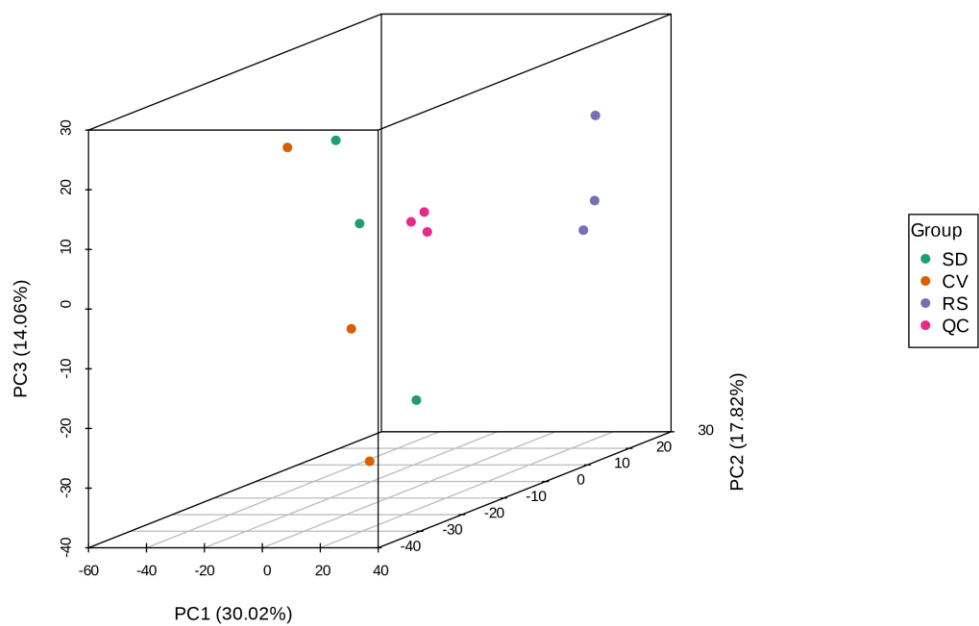

Supplement: Supplementary file 1 [file plants-12-03981-s001.zip › plants-2702487-supplementary/Figure S2. 3D PCA score plots from the mass spectrometry data of the samples in each group and the quality control samples..pdf]

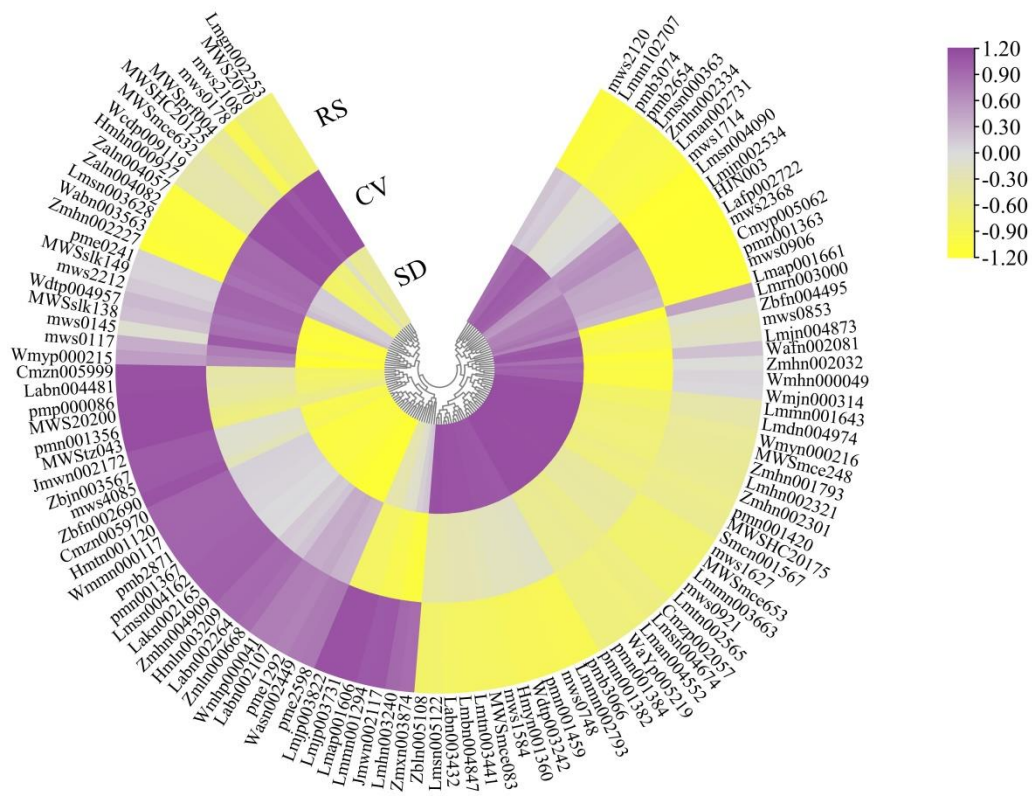

Supplement: Supplementary file 1 [file plants-12-03981-s001.zip › plants-2702487-supplementary/Figure S3. Clustering heatmap of 114 phenolic acids among RS, SD and CV.pdf]

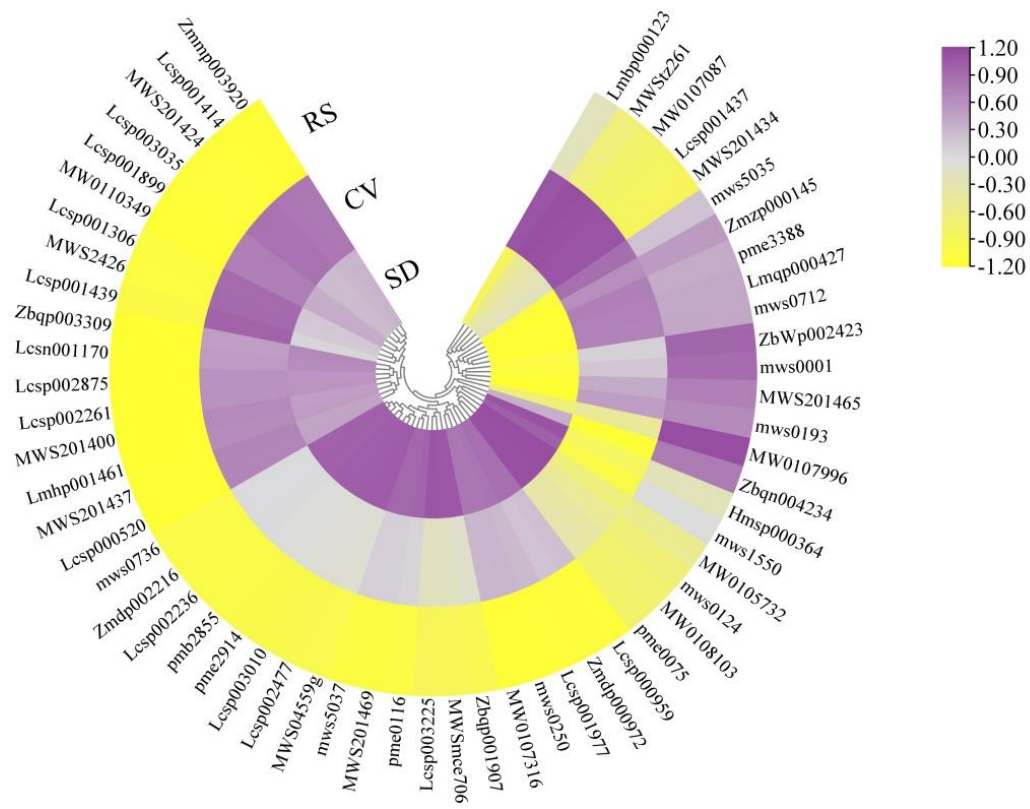

Supplement: Supplementary file 1 [file plants-12-03981-s001.zip › plants-2702487-supplementary/Figure S4. Clustering heatmap of 58 amino acids and derivatives among RS, SD and CV.pdf]

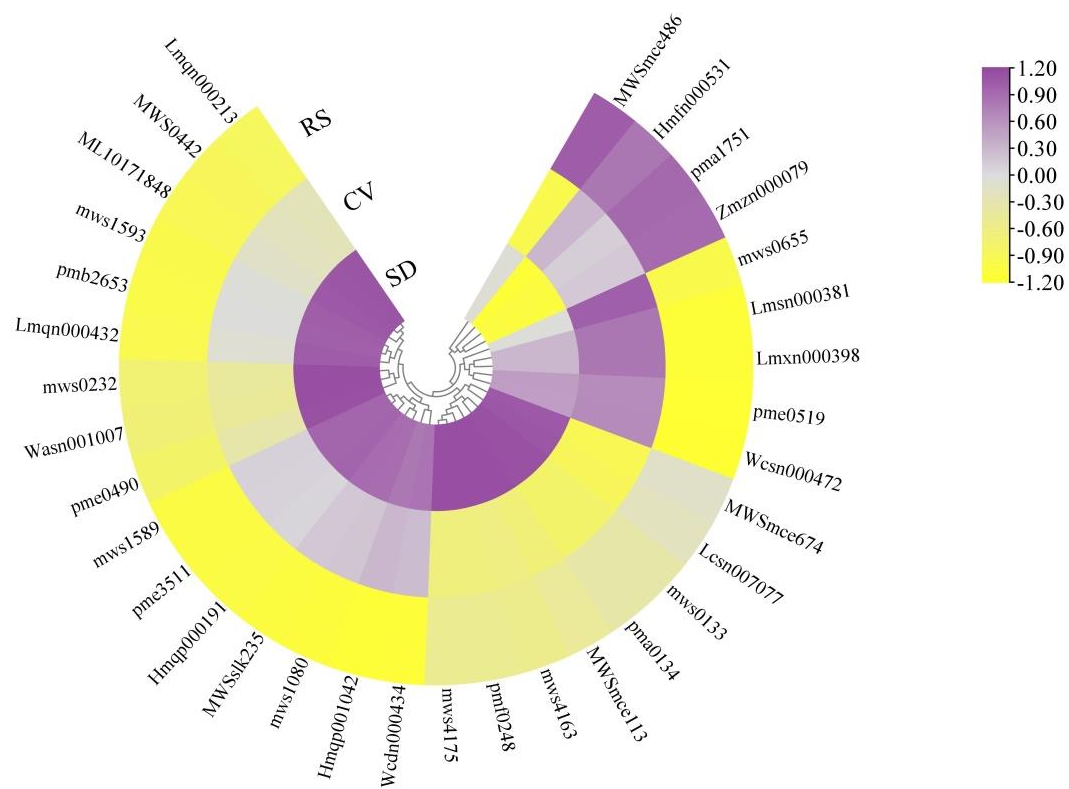

Supplement: Supplementary file 1 [file plants-12-03981-s001.zip › plants-2702487-supplementary/Figure S5. Clustering heatmap of 23 sugars and 10 vitamins among RS, SD and CV.pdf]

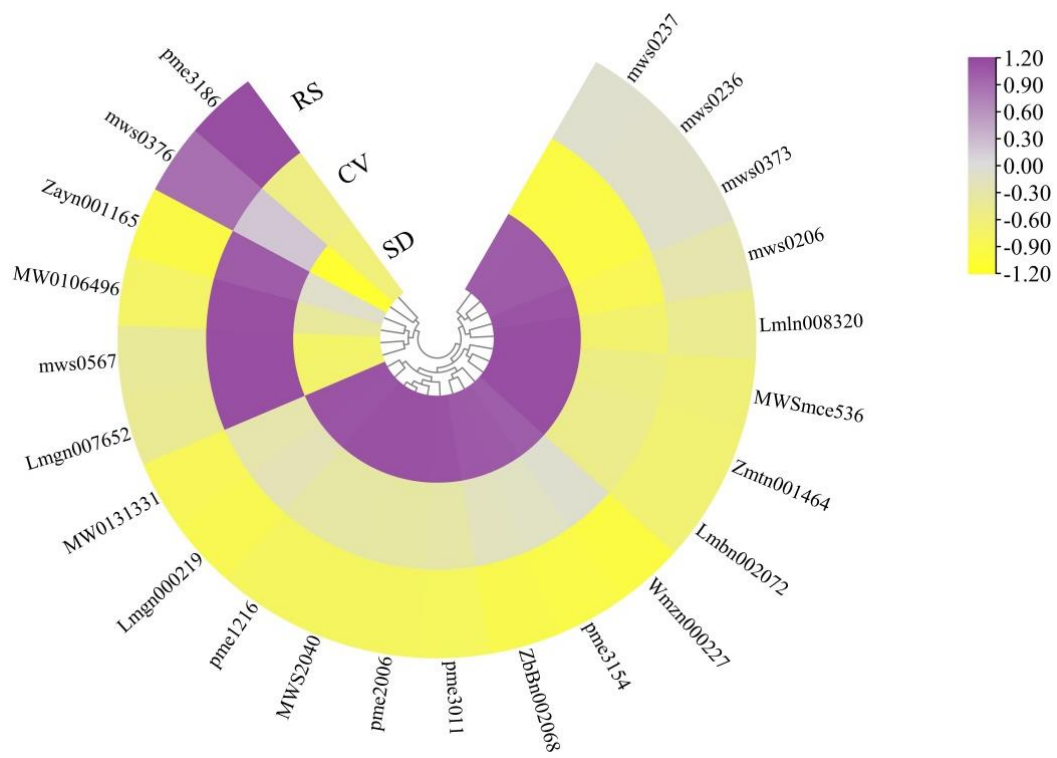

Supplement: Supplementary file 1 [file plants-12-03981-s001.zip › plants-2702487-supplementary/Figure S6. Clustering heatmap of 23 organic acids among RS, SD and CV.pdf]

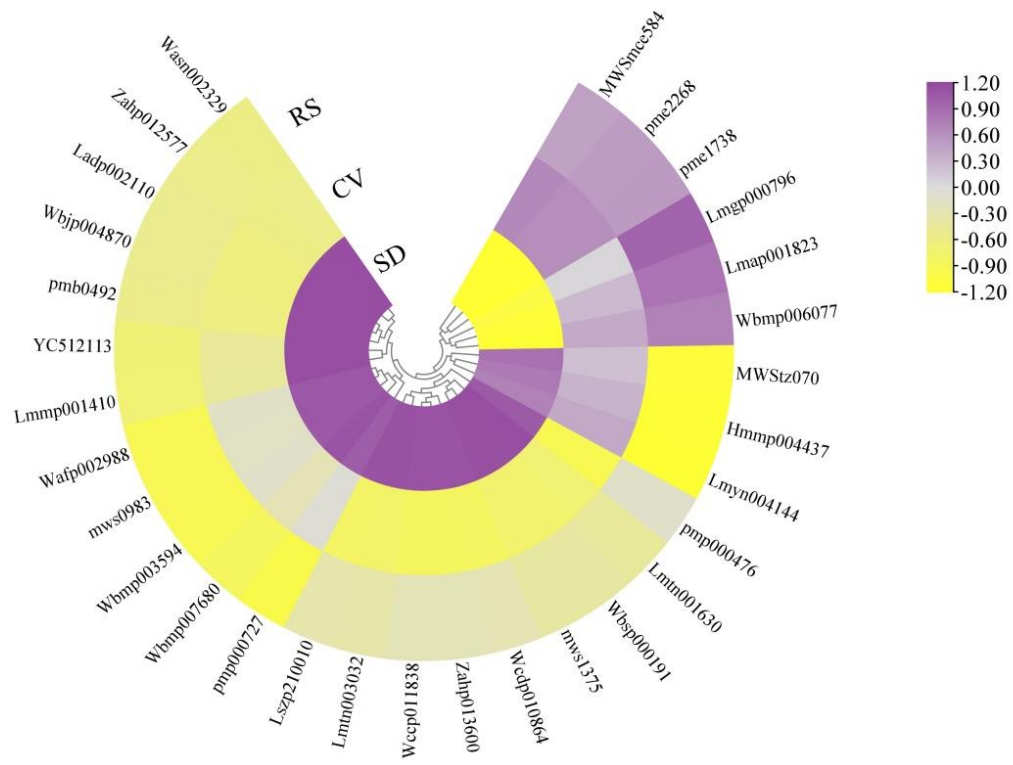

Supplement: Supplementary file 1 [file plants-12-03981-s001.zip › plants-2702487-supplementary/Figure S7. Clustering heatmap of 30 alkaloids among RS, SD and CV.pdf]

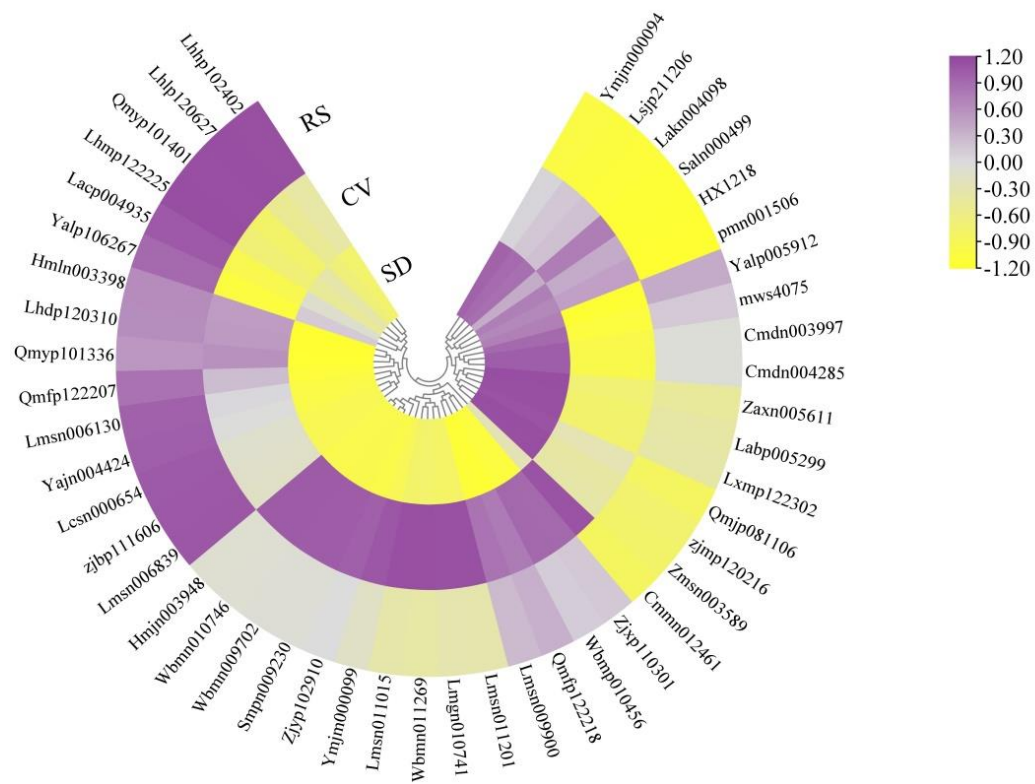

Supplement: Supplementary file 1 [file plants-12-03981-s001.zip › plants-2702487-supplementary/Figure S8. Clustering heatmap of 46 terpenoids among RS, SD and CV.pdf]
